# Effects of Fat Supplementation during Gestation on Reproductive Performance, Milk Composition of Sows and Intestinal Development of Their Offspring

**DOI:** 10.3390/ani9040125

**Published:** 2019-03-28

**Authors:** Xie Peng, Chuan Yan, Liang Hu, Yan Liu, Qin Xu, Ru Wang, Linlin Qin, Cheng Wu, Zhengfeng Fang, Yan Lin, Shengyu Xu, Bin Feng, Yong Zhuo, Jian Li, De Wu, Lianqiang Che

**Affiliations:** Key Laboratory for Animal Disease Resistance Nutrition of the Ministry of Education, Animal Nutrition Institute, Sichuan Agricultural University, Chengdu 611130, China; pengxie2014@foxmail.com (X.P.); yanchuan2013@163.com (C.Y.); huliangsau90@hotmail.com (L.H.); liuyan.2013@foxmail.com (Y.L.); xuqin20143213@163.com (Q.X.); wang.ru@yahoo.com (R.W.); master2015@163.com (L.Q.); 18283581065@163.com (C.W.); fangzhengfeng@hotmail.com (Z.F.); able588@163.com (Y.L.); shengyu_x@hotmail.com (S.X.); fengb123d@163.com (B.F.); zhuoyong@sicau.edu.cn (Y.Z.); lijian522@hotmail.com (J.L.); sow_nutrition@sina.com (D.W.)

**Keywords:** oil, colostrum, reproduction, intestine, sow, piglets

## Abstract

**Simple Summary:**

Nutrition plays a major role in enhancing reproductive efficiency in pigs. Sows mobilize sufficient energy from their body tissue stores for fetal nutritional demands. Dietary fats are used in late gestation and/or lactation diets as sources of energy and essential fatty acids. Our results suggested that fat supplementation during gestation improved colostrum composition and plasma concentration of prolactin at farrowing, associated with altered intestinal morphology and innate immunity in newborn offspring.

**Abstract:**

Various fats are used in swine diets as sources of energy and essential fatty acids. Our aim was to evaluate the effects of fat supplementation during gestation on reproductive performance, milk composition of sows and intestinal development of their offspring. Fifty sows were randomly allocated into two groups receiving the control (CON) and high-fat diets (HF diet) during gestation. After farrowing, all sows received the same lactation diet and were fed ad libitum until weaning at day 20 of lactation. The results showed that being fed the HF diet did not markedly improve the performance of sows and their offspring. However, the HF diet increased (*p* < 0.05) the colostrum contents of protein and no-fat solids, and the plasma concentration of prolactin at farrowing. Moreover, piglets born of sows fed the HF diet had higher (*p* < 0.05) jejunal villous height, as well as deeper (*p* < 0.05) jejunal and colonic crypt depths compared with piglets born of sows fed the CON diet. In addition, piglets born of sows fed the HF diet had markedly increased (*p* < 0.05) mRNA abundances of innate immunity-related genes on toll-like receptor 4 (*TLR-4*), toll-like receptor 9 (*TLR-9*) and myeloid differentiation factor 88 (*MyD88*) in ileum compared with piglets born of sows fed the CON diet. These findings indicated that dietary fat supplementation during gestation did not markedly improve the performance of sows and their offspring, but improved colostrum quality and concentration of prolactin on the day of farrowing, associated with modifications of intestinal morphology and innate immunity of their offspring.

## 1. Introduction

It is widely accepted that fetal nutritional demands are greatly increased during late gestation and lactation, and maternal reserves change to a catabolic condition during this period, particularly when dietary energy supply is insufficient to meet requirements [1]. Dietary fats are used in late gestation and/or lactation diets as sources of energy and essential fatty acids to improve milk yield or fat content [2,3], prevent excessive body reserve mobilization [4] and improve offspring survival rate [5]. However, some researchers reported no significant litter performance and milk production improvement when supplementing dietary fat in late gestation sow diets [6,7], which had been ascribed to the variations in the fat source and quantity, as well as the length of time fat was supplied in the diet [8,9]. In addition, maternal fat supplementation has been shown to induce metabolic disease in offspring, such as insulin resistance, liver dysfunction and high blood pressure [10]. Our recent study showed that fat supplementation during gestation could modulate fetal gene expressions and related signal transduction on intestinal immunity and metabolism [11]. For these studies, however, the biological responses may not only relate to fatty acid supply, but also the greater energy intake when fat supplementation was at higher levels. To our knowledge, the data are limited when it comes to indicating whether dietary isoenergetic intake, but using fat as an energy source during the whole gestation, affects sow performance and their milk composition, as well as intestinal development in offspring.

The objectives of this study were to determine the effects of fat supplementation as part of the energy source during the entire gestation period on reproductive performance, milk composition and endocrine hormones of sows, as well as their offspring’s intestinal morphology and innate immunity.

## 2. Materials and Methods

All procedures of the experiment were approved by the Animal Care and Use Committee of Animal Nutrition Institute, Sichuan Agriculture University, and followed the current laws of animal protection (Ethics Approval Code: SCAUAC201408-3).

### 2.1. Animals and Diets

A total of 50 sows of three to six parities (Landrace × Yorkshire) with similar body conditions (backfat thickness at 17.8 ± 0.60 mm) were used in this experiment. Sows were evenly allocated by parity into two groups (25 sows/group). The sows were artificially inseminated with semen from a pool of Duroc boars. They were housed in individual gestation stalls (2.13 × 0.61 m). The control sows were fed a control (CON) diet and the sows in the high-fat (HF) group were fed a diet containing 2% soybean oil. The HF diet was formulated by mainly reducing the corn but increasing the wheat bran and soybean oil contents. The experimental diets were formulated to meet or exceed the nutrient requirements of gestating and lactating sows as recommended by National Research Council NRC (2012) (Table 1 and Table 2). Daily feed intakes were adjusted to ensure a similar intake of nutrients among diets during gestation (Table 3). Gestation diets were supplied twice a day (07:00 and 17:30). On day 107 of gestation, sows were transported to farrowing stalls and individually fed. After farrowing, all sows received the same standard lactation diet. During the 24 h post-farrowing, litter sizes were adjusted to nine or 10 piglets by cross-fostering within the same treatment. The lactation diet was supplied three times a day (08:00, 11:30 and 17:30) and started at 2.0 kg/day, then increased by 0.5 kg/day during the first week as previously reported [12]. Afterwards, sows had free access to the diet until they were weaned on day 20 of lactation. Sows were provided ad libitum access to water during the entire experimental trial. The temperature was maintained at 20~22 °C in the gestating house, while it was 24~26 °C in the lactating house, and supplemental heat was provided to piglets with heat lamps (250 W).

### 2.2. Measurements

The backfat thickness of sows was measured at 65 mm to the left side of the dorsal mid-line at the level of the last rib (P2) using an ultrasonic device (Renco Lean-Meatier^®^; Renco Corporation, Minneapolis, MN, USA) and recorded at days 1 and 90 of gestation, farrowing and day 20 of lactation. After birth, piglets were classified as born alive or stillborn as described previously [13], and the number of low body weight (BW) piglets (<1.0 kg) was recorded. Piglets were weighed individually at parturition and day 20 of lactation. Daily feed intake during lactation was recorded.

### 2.3. Sample Collection

Blood samples were sampled by ear venipuncture from a random subset of sows (n = 8) at day 90 of gestation and farrowing. The same subset of sows was bled at each time period. Approximately 5 mL of blood per sow was collected into heparinized tubes and centrifuged at 3000× *g* at 4 °C for 15 min; plasma was separated and immediately stored at −80 °C for later analysis. Immediately after parturition, colostrum samples (10 mL) were collected after the sow was injected with 1 mg of oxytocin (Hangzhou Animal Medicine Factory, Hangzhou, China) in the ear vein. Likewise, milk samples on days 10 and 20 of lactation were collected. Colostrum and milk samples were immediately frozen at −20 °C until analysis.

After farrowing, eight piglets (one male piglet from each group of eight litters) whose BW was closest to the average BW of their litter was selected and anesthetized with an intravenous injection of Zoletil 50 at a dose of 0.1 mg/kg BW before jugular exsanguination as previously reported [14]. After the abdomen was exposed, the heart, spleen, pancreas, kidney and liver were quickly removed and weighed. The weight of small intestine was measured after the removal of luminal contents. Relative small intestine, heart, spleen, pancreas, kidney and liver weights were expressed as the ratio to body weight (g/kg). The jejunal, ileal and colonic samples (approximately 2 cm in length) were stored in 4% paraformaldehyde solution for histological measurement. Another portion of the ileum (approximately 2 cm in length) was snap-frozen and then stored at −80 °C until analysis.

### 2.4. Histological Measurements

By referring to our previous study [15], the intestinal tissues preserved in the 4% paraformaldehyde solution were embedded in paraffin. Each tissue sample was used to prepare five slides and each slide had three sections (5 µm thick), which were stained with hematoxylin and eosin for intestinal morphology measurement by 20 intact well-oriented crypt-villus units in each section (Optimus software version 6.5, Media Cybergenetics, North Reading, MA, USA).

### 2.5. Chemical Analysis

The milk composition was analyzed using an automatic milk analyzer (Milk-Y Way-CP2, Beijing, China). The plasma metabolites from sows (glucose, triacylglycerol, urea) were determined using specific assay kits (Nanjing Jiancheng Bioengineering Institute, Nanjing, China) according to the manufacturer’s instructions. Plasma concentrations of progesterone, prolactin and leptin in sows were determined using commercial ELISA kits (Nanjing Jiancheng Bioengineering Institute, Nanjing, China) according to the manufacturer’s protocol. There was less than 5% variation of intra-assay and inter-assay coefficients for each assay.

### 2.6. Gene Expression

As in our previous study [15], total RNA was extracted from frozen ileal samples using TRIzol reagent (TaKaRa Biotechnology Co. Ltd., Dalian, China) according to the manufacturer’s instructions. The quality and purity of the RNA samples were assessed by agarose gel (1.0%) electrophoresis and nucleic acid analyzer (A260/A280, Beckman DU-800; Beckman Coulter, Inc., Brea, CA, USA), respectively. A commercial reverse transcription (RT) kit (TaKaRa Biotechnology Co. Ltd., Dalian, China) was used in the synthesis of cDNA. cDNA was amplified using a real-time PCR system (ABI 7900HT, Applied Biosystems, USA). The reaction mixture (10 µL) contained 5.0 µL of freshly SYBR^®^ Premix Ex Taq II (Tli RNaseH Plus), 0.4 µL forward primer (10 μM), 0.4 µL reverse primer (10 μM), 0.2 µL ROX Reference Dye (50×), 1.0 µL cDNA and 3.0 µL double distilled water. Cycling conditions were 95 °C for 30 s, followed by 40 cycles of 95 °C for 5 s and 60 °C for 34 s. At the end of amplification, melting curves were performed to identify the specificity of PCR-amplified product. The 2^−ΔΔCt^ method was used to process the real-time PCR results as described previously [16]. Evaluation of *β-actin* C_T_ values across samples showed that there was no effect of treatment on *β-actin* expression (i.e., *β-actin* was acceptable as a housekeeping gene). All samples were measured in triplicate. The sequences of the primers are listed below. *TLR-2* forward TCGAAAAGAGCCAGAAAACCAT and reverse CTTGCACCACTCGCTCTTCA; *TLR-4*, forward AGAAAATATGGCAGAGGTGAAAGC and reverse CTTCGTCCTGGCTGGAGTAGA; *TLR-9*, forward AATCCAGTCGGAGATGTTTGCT and reverse GACCGCCTGGGAGATGCT; *MyD88*, forward GTGCCGTCGGATGGTAGTG and reverse TCTGGAAGTCACATTCCTTGCTT; *TRAF-6*, forward GCTGCATCTATGGCATTTGAAG and reverse CCACAGATAACATTTGCCAAAGG; *NF-κB*, forward TGCTGGACCCAAGGACATG and reverse CTCCCTTCTGCAACAACACGTA; *IL-1β*, forward TCTGCCCTGTACCCCAACTG and reverse CCAGGAAGACGGGCTTTTG; *IL-6*, forward GATGCTTCCAATCTGGGTTCA and reverse CACAAGACCGGTGGTGATTCT; *SIGIRR*, forward ACCTGGGCTCCCGAAACTAC and reverse GTCATCTTCTGACACCAGGCAAT; *β-Actin*, forward GGCGCCCAGCACGAT and reverse CCGATCCACACGGAGTACTTG.

### 2.7. Statistical Analysis

The individual sow and piglet were used as the experimental unit for all response variables in the model, which included diet (CON or HF) as the main effect. Data were analyzed by an independent-samples *t*-test using SPSS 21.0 (IBM SPSS Company, Chicago, IL, USA). Piglet performance during the first day after birth was analyzed for all the piglets nursed during that day. Litter size after cross-fostering was used as a covariate for piglet and litter performance from days 1 to 20 of lactation. The sow was used as the experimental unit for the analysis of data of reproductive performance, plasma parameters, colostrum and milk composition, whereas the piglet was used as the experimental unit for the analysis of data involving piglet performance, gut morphology, relative organ weights, and gene expression in the ileum. All values were expressed as means with their standard errors (SEM). Differences were considered as significant when *p* < 0.05, whereas 0.05 < *p* < 0.10 was considered a tendency.

## 3. Results

### 3.1. Performance of Sows and Their Offspring

At the onset of the experiment (day 1 of gestation), sow backfat thickness was similar in both treatments. Sow backfat thickness at day 90 of gestation, farrowing and day 20 of lactation were not influenced by dietary supplementation with fat (Table 4). However, dietary fat supplementation increased backfat gain during days 1 to 90 of gestation (*p* < 0.05). The total number of pigs born, born alive, stillborn, as well as the number of low BW piglets, individual birth weights and mean litter weight at birth were unaffected between the two dietary treatments. In addition, average daily feed intake during lactation of sows did not differ between treatments.

After cross-fostering, litter size was similar in both treatments. No differences were observed for the number of piglets at weaning per litter, the pre-weaning survival, the average pig weight and litter weight at weaning between treatments (data not shown).

### 3.2. Plasma Hormones and Metabolites

As shown in Table 5, sows subjected to the HF treatment had greater (*p* < 0.05) plasma prolactin concentration when compared to sows fed the CON treatment at farrowing. However, no differences were observed for plasma progesterone concentration at day 90 of gestation and leptin concentration at farrowing between groups. In addition, there was no observed significant dietary treatment effects on the plasma triacylglycerol, urea and glucose concentration at farrowing.

### 3.3. Colostrum and Milk Composition

The composition of the sow colostrum and milk are shown in Table 6. The protein and no-fat solids concentrations in colostrum were significantly greater (*p* < 0.05) in sows from the HF group when compared to sows from the CON group. There were no significant protein, fat, lactose or no-fat solids concentration differences from the milk at days 10 and 20 of lactation from sows fed the two dietary treatments.

### 3.4. Gut Morphology and Relative Organ Weights

As shown in Table 7, in the jejunum, piglets born of sows fed the HF diet exhibited greater (*p* < 0.05) villus height and crypt depth compared with piglets born of sows fed the CON diet. In the colon, however, piglets born of sows fed the HF diet exhibited deeper (*p* < 0.05) crypt depth and a lower (*p* < 0.05) ratio of villus height to crypt depth compared with piglets born of sows fed the CON diet. There were no significant effects of dietary treatment on relative organ weights of the small intestine, heart, spleen, pancreas, kidney and liver in piglets (data not shown).

### 3.5. Gene Expression in the Ileum

As shown in Table 8, piglets born of sows fed the HF diet had markedly increased mRNA expression of *TLR-4*, *TLR-9* and *MyD88* (*p* < 0.05) in the ileum compared with piglets born of sows fed the CON diet. However, there was no observed significant dietary treatment effects on the mRNA expression of *TLR-2*, *TRAF-6*, *NF-κB*, *IL-1β*, *IL-6* and *SIGIRR* in the ileum.

## 4. Discussion

Results from the present study showed that fat supplementation during gestation did not markedly affect reproductive performance. This result is in agreement with a previous study [17] which showed that the isoenergetic replacement of starch with palm oil during gestation had no significant effect on litter performance. The similar results had been also observed in our recent study [18]. Previous studies have shown that sows fed supplemental fat at more than 5% improved the litter growth performance [2,8], and only a few studies have shown a significantly positive effect on breast-feeding performance by the addition of less than 5% fat [19,20]. In addition, the backfat thickness and metabolic characteristics such as triacylglycerol, urea and glucose were not markedly altered by fat supplementation, which may be ascribed to the dietary isoenergetic intake. However, sows fed the HF diet in gestation had greater sow backfat gain during days 1 to 90 of gestation compared with sows fed the CON diet, which may be due to the higher relative fat deposition efficiency of soybean oil than that of cereal grains. Furthermore, prolactin is one of the main hormones involved in the initiation and maintenance of milk production in mammals [21]. We observed that sows fed the HF diet had greater plasma prolactin concentration than sows fed the CON diet at farrowing, suggesting that dietary fat supplementation during gestation had a beneficial effect on the lactating capability of sows, as indicated by the increased milk yield in sows fed the fat-supplemented diet [3,22].

The milk composition had an important influence on the growth and development of suckling piglets [4]. The current findings showed that fat supplementation during gestation increased the colostral protein concentration. Similarly, a previous study showed that the isoenergetic replacement of maize starch with soy oil in sow lactation diets tended to have greater protein concentration in milk [23], which may be attributed to the development of the mammary gland [24] because fatty acids are required for mammary ductal and alveolar structure development [25]. Data from lambs indicated that dietary polyunsaturated fatty acids stimulate mammary growth through increasing the expression of prolactin receptor in mammary parenchyma and growth hormone receptor in liver [26], suggesting a beneficial effect of dietary fat supplementation on improving colostrum composition. In addition, the higher colostral protein concentration in sows fed the HF diet may suggest higher immunoglobulin content in the colostrum, which would provide immediate immune protection to newborn piglets [27,28]. However, the inconsistent results showed that the fat concentration in colostrum and milk was not markedly increased by fat supplementation during gestation, which may be related to the dosage and sources of fat [9]. Furthermore, the limited number of sows used in our experiment cannot be ignored.

Fetuses receive nutrients from mothers via the placenta, while postnatal pups must take up nutrients from food via the small intestine; therefore, small intestinal development during gestation period plays an important role [14]. Villous height shortening may imply that there is a decreased surface area for nutrient absorption, while a deeper crypt indicates a faster turnover of new villous cells [29]. Previous study has indicated that maternal nutrition would affect the intestinal development and function of offspring [30]. It is well known that fat supplementation during gestation and lactation affects the fatty acid composition of milk and the tissues of piglets. Boudry et al. (2009) observed a modified fatty acid composition and structure of the ileum of piglets when supplementing 3.0% linseed oil to the sow diet during gestation and lactation [31]. Relative to piglets born of sows fed the CON diet, in the present study, piglets born of sows fed the HF diet had greater jejunal villous height and deeper crypt depth in the jejunum and colon. Supportively, our studies in sows have showed that maternal soybean oil intake improves not only intestinal morphology, but also enzyme activity and growth factors [11,14]. Therefore, it may be concluded that fat supplementation during gestation may improve the intestinal development of offspring.

The intestine is the largest immunological organ in the body, and as such is the location for the majority of lymphocytes and immune effector cells with pattern-recognition receptors [32]. It has been demonstrated that toll-like receptors (TLRs) are typical pattern-recognition receptors in mediating mucosal innate host defense to maintain mucosal and commensal homeostasis [33]. The *TLR4*-*MyD88*-*NF*-*κB* signal pathway is involved in the inflammatory response [34]. Our previous study showed that maternal fat supplementation during gestation altered the fetal intestinal immune response, signal transduction and metabolism [11]. In this study, there were increased gene expressions for *TLR-4*, *TLR-9* and *MyD88* in the ileum of piglets born of sows fed the HF diet, suggesting the modulating effects of fat supplementation during gestation on the intestinal innate immunity of newborn piglets. Similarly, a previous study reported that maternal fat supplementation could transfer fatty acids to fetus and regulate the fetal intestinal physiology and function through altering the phospholipid fatty acids composition of the intestinal membrane [30].

## 5. Conclusions

In conclusion, our results indicated that fat supplementation during gestation had no significant effect on the performance of sows and their offspring, but it improved colostrum composition and plasma concentration of prolactin at farrowing, associated with altered intestinal morphology and innate immunity in newborn offspring. The results provide some evidence that fat supplementation may improve the lactating capability in sows and enhance the immune function of newborn piglets.

## Figures and Tables

**Table 1:** Composition and nutrient levels of gestation diets (air dry basis, %).

Item	Days 1–90 of Gestation		Day 91 of Gestation to Parturition	
	CON	HF	CON	HF
Ingredient, %				
Corn, 7.8% CP	63.60	59.51	64.11	59.38
Soybean meal, 44.2% CP	5.00	5.00	11.67	12.89
Wheat bran	28.76	30.76	20.66	22.18
Soybean oil	-	2.00	-	2.00
L-Lysine·HCl, 75%	0.10	0.11	0.15	0.14
L-Threonine	0.01	0.02	0.05	0.05
Calcium carbonate	0.61	0.64	0.96	0.96
Dicalcium phosphate	0.97	1.01	1.45	1.45
Salt	0.40	0.40	0.40	0.40
Choline chloride (50%)	0.15	0.15	0.15	0.15
Vitamins and trace minerals premix^1^	0.4	0.4	0.4	0.4
Total	100.00	100.00	100.00	100.00
Nutrient levels				
NE, Mcal/kg	2.22	2.30	2.24	2.31
CP, %	11.10	11.08	13.00	13.38
EE, %	3.47	5.37	3.29	5.17
NDF, %	17.30	17.80	14.90	15.32
Ca, %	0.47	0.49	0.72	0.72
Available P, %	0.24	0.25	0.36	0.36
Lys, %	0.51	0.52	0.69	0.71
Met, %	0.18	0.18	0.21	0.21
Thr, %	0.40	0.41	0.52	0.54
Trp, %	0.12	0.12	0.14	0.15
Val, %	0.51	0.50	0.59	0.61

CON, control group; HF, high-fat group; CP, crude protein; NE, net energy; EE, ether extract; NDF, neutral detergent fiber.^1^ Trace minerals and vitamins premix provided per kilogram of diet: Zn (as zinc sulfate), 100 mg; Cu (as copper sulfate), 30 mg; Fe (as ferrous sulfate), 100 mg; Mn (as manganese sulfate), 40 mg; I (as potassium iodide), 0.25 mg; Se (as sodium selenite), 0.25 mg; Cr (as chromium picolinate), 0.3 mg; 25-hydroxycholecalciferol, 0.07 mg; vitamin A, 2 mg; vitamin B_6_, 14 mg; vitamin E, 30 mg; vitamin C, 100 mg; biotin, 0.1 mg; folic, acid 2.5 mg; carnitine, 46 mg.

**Table 2:** Composition and nutrient levels of lactation diet (air dry basis, %).

Item	Lactation
Ingredient, %	
Corn, 7.8% CP	59.86
Soybean meal, 44.2% CP	17.68
Fishmeal	2.00
Extruded soybean	6.00
Wheat	3.59
Soybean bran	5.00
Soybean oil	2.00
L-Lysine·HCl, 75%	0.25
L-Threonine	0.09
DL-Methionine	0.05
Calcium carbonate	0.87
Dicalcium phosphate	0.93
Salt	0.40
Potassium chloride	0.50
Choline chloride (50%)	0.15
Vitamins and trace minerals premix^1^	0.63
Total	100.00
Nutrient levels	
ME, Mcal/kg	3.41
CP, %	17.20
EE, %	6.10
NDF, %	11.00
Ca, %	0.90
Available P, %	0.45
Lys, %	1.00
Met, %	0.27
Thr, %	0.63
Trp, %	0.18

ME, metabolizable energy. ^1^ Trace minerals and vitamins premix provided per kilogram of diet: Zn (as zinc sulfate), 100 mg; Cu (as copper sulfate), 30 mg; Fe (as ferrous sulfate), 100 mg; Mn (as manganese sulfate), 40 mg; I (as potassium iodide), 0.25 mg; Se (as sodium selenite), 0.25 mg; Cr (as chromium picolinate), 0.3 mg; 25-hydroxycholecalciferol, 0.07 mg; vitamin A, 2 mg; vitamin B_6_, 14 mg; vitamin E, 30 mg; vitamin C, 100 mg; biotin, 0.1 mg; folic acid, 2.5 mg; carnitine, 46 mg.

**Table 3:** Daily feed intakes during gestation of sows.

Day of Gestation	CON	HF
Days 1~30, kg/days	2.13	2.06
Days 31~60, kg/days	2.38	2.31
Days 61~90, kg/days	2.63	2.56
Day 91~farrowing, kg/days	3.01	2.93

CON, control group; HF, high-fat group.

**Table 4:** Effects of dietary fat supplementation during gestation on backfat thickness and reproductive performance of sows.

Items	CON	HF	*p*-Value
Sow backfat thickness, mm			
Day 1 of gestation	17.1 ± 1.05	17.9 ± 0.64	0.46
Day 90 of gestation	17.9 ± 1.02	19.8 ± 0.59	0.10
Farrowing day	18.7 ± 1.38	20.1 ± 0.62	0.37
Weaning day	17.5 ± 1.26	19.0 ± 0.60	0.34
Backfat thickness changes, mm			
Days 1–90 of gestation	0.81 ± 0.44 ^a^	1.91 ± 0.54 ^b^	<0.05
Day 1 of gestation to farrowing	1.61 ± 0.56	2.15 ± 0.64	0.41
Farrowing day to weaning day	−1.26 ± 0.34	−1.02 ± 0.47	0.60
Litter size at birth, No/litter			
Total born	12.11 ± 0.45	12.74 ± 0.53	0.32
Born alive	11.31 ± 0.44	12.00 ± 0.50	0.31
Stillborn piglets	0.79 ± 0.20	0.74 ± 0.25	0.52
Piglets < 1.0 kg	1.42 ± 0.36	0.96 ± 0.35	0.20
Mean litter weight at birth, kg	15.45 ± 0.68	16.95 ± 0.85	0.30
Piglet mean BW at birth, kg	1.37 ± 0.05	1.41 ± 0.03	0.71
ADFI during lactation, kg	5.09 ± 0.17	5.46 ± 0.22	0.18

CON, control group; HF, high-fat group; BW, body weight; ADFI, average daily feed intake. Values are mean ± standard error, n = 25 for each group.

**Table 5:** Effects of dietary fat supplementation during gestation on plasma hormone and metabolite concentrations of sows (ng/mL).

Items	CON	HF	*p*-Value
Day 90 of gestation			
Progesterone	51.77 ± 6.52	54.81 ± 7.24	0.69
Farrowing			
Prolactin	262.00 ± 31.92 ^a^	432.70 ± 41.81 ^b^	<0.01
Leptin	28.80 ± 4.68	35.62 ± 6.78	0.33
Triacylglycerol	0.21 ± 0.03	0.29 ± 0.05	0.10
Urea	3.72 ± 0.31	3.97 ± 0.44	0.65
Glucose	5.34 ± 0.21	5.20 ± 0.31	0.66

CON, control group; HF, high-fat group. Values are mean ± standard error, n = 8 for each group. ^a,b^ Mean values within a row with unlike superscript letters are significantly different (*p* < 0.05).

**Table 6:** Effects of dietary fat supplementation during gestation on composition of colostrum and milk.

Items	CON	HF	*p*-Value
Colostrum			
Fat, %	3.88 ± 0.52	3.56 ± 0.43	0.66
No-fat solids, %	15.53 ± 1.92 ^a^	22.90 ± 2.01 ^b^	<0.01
Protein, %	5.85 ± 0.75 ^a^	8.79 ± 0.82 ^b^	<0.01
Lactose, %	9.36 ± 1.19	12.11 ± 1.60	0.11
Milk at day 10 of lactation			
Fat, %	5.71 ± 0.34	6.69 ± 0.94	0.32
No-fat solids, %	10.04 ± 0.24	9.83 ± 0.52	0.73
Protein, %	3.74 ± 0.09	3.76 ± 0.21	0.92
Lactose, %	5.49 ± 0.22	5.76 ± 0.42	0.60
Milk at day 20 of lactation			
Fat, %	5.40 ± 0.32	6.35 ± 0.69	0.35
No-fat solids, %	9.50 ± 0.86	10.43 ± 0.46	0.44
Protein, %	3.52 ± 0.32	3.89 ± 0.18	0.42
Lactose, %	5.27 ± 0.55	5.83 ± 0.38	0.49

CON, control group; HF, high-fat group; no-fat solids mainly consisted of lactose, protein and minerals. Values are mean ± standard error, n = 8 for each group. ^a,b^ Mean values within a row with unlike superscript letters are significantly different (*p* < 0.05).

**Table 7:** Effects of dietary fat supplementation during gestation on intestinal morphology of piglets.

Items	CON	HF	*p*-Value
Jejunum			
Villous height, μm	717 ± 42 ^a^	923 ± 68 ^b^	0.02
Crypt depth, μm	76 ± 3 ^a^	88 ± 2 ^b^	0.01
VCR	9.60 ± 0.53	10.67 ± 0.86	0.29
Ileum			
Villous height, μm	610 ± 57	567 ± 53	0.58
Crypt depth, μm	75 ± 5	86 ± 7	0.29
VCR	8.29 ± 0.72	6.81 ± 0.66	0.15
Colon			
Villous height, μm	196 ± 9	176 ± 13	0.24
Crypt depth, μm	32 ± 2 ^a^	41 ± 1 ^b^	<0.01
VCR	6.53 ± 0.60 ^a^	4.40 ± 0.40 ^b^	0.01

CON, control group; HF, high-fat group; VCR, villous height to crypt depth ratio. Values are mean ± standard error, n = 8 for each group. ^a,b^ Mean values within a row with unlike superscript letters are significantly different (*p* < 0.05).

**Table 8:** Effects of dietary fat supplementation during gestation on gene expression in the ileum of piglets.

Items	CON	HF	*p*-Value
*TLR-2*	1.00 ± 0.10	0.81 ± 0.12	0.61
*TLR-4*	1.00 ± 0.08 ^a^	1.48 ± 0.14 ^b^	0.04
*TLR-9*	1.00 ± 0.10 ^a^	1.40 ± 0.08 ^b^	0.03
*MyD88*	1.00 ± 0.06 ^a^	1.22 ± 0.07 ^b^	0.04
*TRAF-6*	1.00 ± 0.05	0.89 ± 0.10	0.42
*NF-κB*	1.00 ± 0.10	1.12 ± 0.10	0.46
*IL-1β*	1.00 ± 0.11	0.84 ± 0.13	0.50
*IL-6*	1.00 ± 0.09	1.23 ± 0.11	0.19
*SIGIRR*	1.00 ± 0.10	1.22 ± 0.09	0.21

CON, control group; HF, high-fat group; TLR, Toll-like receptor; MyD88, myeloid differentiation factor 88; TRAF-6, TNF receptor-associated factor 6; NF-κB, Nuclear factor kappa B; IL, interleukin; SIGIRR, single Ig IL-1-related receptor. Values are mean ± standard error, n = 8 for each group. ^a,b^ Mean values within a row with unlike superscript letters are significantly different (*p* < 0.05).
